# Supplementary material for: Identification of photoperiod-induced specific miRNAs in the adrenal glands of Sunite sheep (Ovis aries)
Source: Front Vet Sci. 2022 Jul 22;9:888207. doi: 10.3389/fvets.2022.888207 (PMC9354845; doi:10.3389/fvets.2022.888207)
Supplement: Supplementary file 1 [file Data_Sheet_1.ZIP › Supplementary file/miRDeep2 file.zip!/miRDeep2 file/Novel_Structure/SP42A2/Novel_419.pdf]

Provisional ID	:	NC_019478.2_36778
Score total	:	0
Score for star read(s)	:	-1.3
Score for read counts	:	0
Score for mfe	:	-0.3
Score for randfold	:	1.6
Score for cons. seed	:	
Total read count	:	70
Mature read count	:	70
Loop read count	:	0
Star read count	:	0

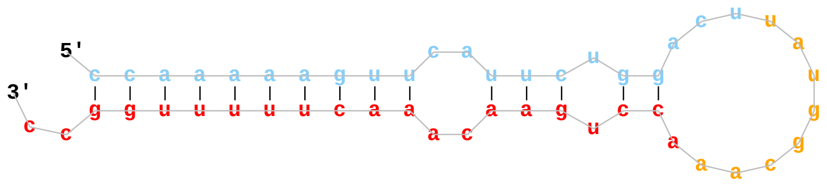

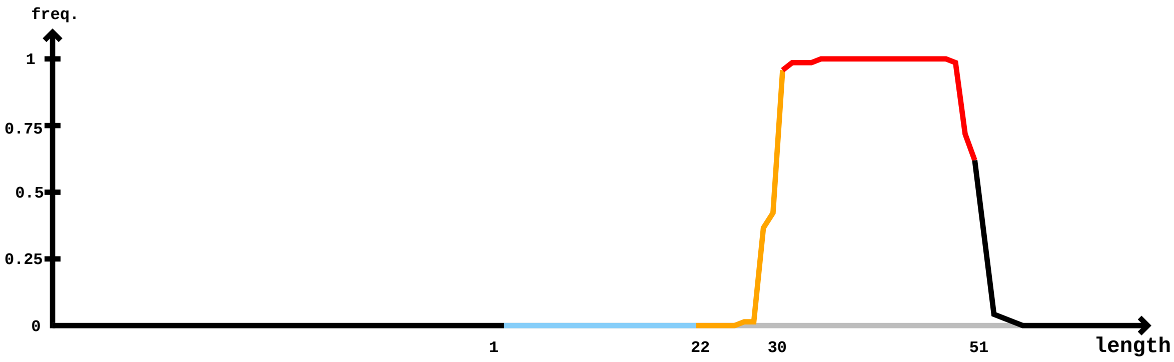

Star Mature

```
5'- uguugugagaaaaccaccaccuguuuuguaaaaguaucaggauggccaaaaaguucauucuggacuuauggcaaaccugaacaaacuuuuuggccaacucaaaaguuuuuug   -3'    exp
    .......(((((((.....((((..............)))).(((((((((((((..(((.((............)).)))..)))))))))))))........))))))).    reads       mm          sample
    ......................................................................gcaaaccugaacaaacuuuuug....................    1           0           seq
    ........................................................................aaaUcugaacaaacuuuuugg...................    19          1           seq
    ........................................................................aaaccugaacaaacuuuuuggU..................    3           1           seq
    ........................................................................aaaAcugaacaaacuuuuuggc..................    2           1           seq
    ........................................................................aaaccugaacaaacuuuuuggcA.................    1           1           seq
    .........................................................................aaUcugaacaaacuuuuuggc..................    2           1           seq
    .........................................................................aaUcugaacaaacuuuuuggcc.................    2           1           seq
    ..........................................................................accugaacaaacCuuuuggcc.................    1           1           seq
    ..........................................................................aUcugaacaaacuuuuuggcc.................    25          1           seq
    ..........................................................................aUcugaacaaacuuuuuggcca................    11          1           seq
    ..........................................................................accugaacaaacCuuuuggccaac..............    1           1           seq
    ...........................................................................ccugaacaaCcuuuuuggcc.................    1           1           seq
    ...........................................................................Ucugaacaaacuuuuuggccaa...............    1           1           seq
    ..............................................................................gaacGaacuuuuuggccaacu.............    1           1           seq
```
